# Supplementary material for: Action learning and public health pedagogy: Student reflections from an experiential public health course
Source: Front Public Health. 2023 Mar 28;11:1128705. doi: 10.3389/fpubh.2023.1128705 (PMC10086179; doi:10.3389/fpubh.2023.1128705)
Supplement: Supplementary file 1 [file Data_Sheet_1.ZIP › Supplementary Material Presentation/Appendix A - Assignment Instructions 1.pdf]

Year	Assignment Instructions
2008	The reflection paper is intended to help you process and apply some of the course readings to what you are doing in your group project as well as to your past work experiences. The paper should be 2-3 pages long (1 ½ -spacing).<br><br>The paper should be in the first person, and should present some of your reflections on the ideas raised in at least two of the course readings. The paper should be a synthesis of critical ideas from the course readings and questions the readings generated for you. You are expected to reflect on the themes/points in the readings and how they may be applied to your current community project or past work. Please also include reflections on one of the public health code of ethics guidelines.
2009-2013	Please reflect on how your project for the Community Health Program Planning* course did (or did not) meet the Yale School of Public Health practice guidelines [See Appendix B]. The paper should be 1-2 pages long (single spaced).<br><br>The paper should be in the first person, and should present some of your reflections on the learning experiences and tasks of your project within the context of specified guidelines. Identify any guidelines which you may not have met, and guidelines which were most strongly emphasized in your project.
2014-2015	Please reflect on how your project for the Community Health Program Planning* course did (or did not) meet the Yale School of Public Health practice guidelines [See Appendix B]. The paper should be 1-2 pages long (single spaced).<br><br>The paper should be in the first person, and should present some of your reflections on the learning experiences and tasks of your project within the context of specified guidelines. Identify any guidelines which you may not have met, and guidelines which were most strongly emphasized in your project. Reflect on key challenges and learning opportunities you have had during this process.
2016-2017	Please identify two to three MPH Core competencies (see attached) and two to three cross-cutting competencies [See Appendix B] that you have addressed in your project for the Practice-Based Community Health Research course. Reflect on how your project has enhanced those competencies. Orient some of the tasks of your project within the context of the core and cross-cutting competencies you identify, and describe some of the challenges and learning opportunities you have had during this process, particularly as they relate to the core and cross-cutting competencies.
2018	Please identify two to three MPH Core competencies [See Appendix B] that you have addressed in your project for the Practice-Based Community Health Research course. Reflect (with specific examples) on how your project has enhanced those competencies. Orient some of the tasks of your project within the context of the core competencies you identify, and describe some of the challenges and learning opportunities you have had during this process, particularly as they relate to the core competencies.<br><br>In addition to the core competencies, please choose one or two leadership, communication, or collaborative skills that you have strengthened or developed during this course and describe (with examples) how the class has affected those skills.

*The course was renamed as Practice-Based Community Health Research in 2015
