# Supplementary material for: Action learning and public health pedagogy: Student reflections from an experiential public health course
Source: Front Public Health. 2023 Mar 28;11:1128705. doi: 10.3389/fpubh.2023.1128705 (PMC10086179; doi:10.3389/fpubh.2023.1128705)
Supplement: Supplementary file 1 [file Data_Sheet_1.ZIP › Supplementary Material Presentation/Appendix C - Quotes.pdf]

Themes & Subthemes	Exemplar Quote
**Turning theory into practice**<br>Hands on experience<br>Opportunity to do meaningful work<br>Relevance to future career<br>Connecting big data to lived experiences	*"One of the most valuable aspects of this project was being able to see firsthand the dynamics at work in a community health center. Conducting informational interviews and observing the resources available to [community organization] provided insight in a way that reading an article from Health Affairs cannot touch." (Student 2, 2014 Cohort)*
**Navigating the complex environment of public health practice**<br>Power dynamics in public health research and practice<br>Understanding differing value systems<br>Influence of social and political factors<br>Balancing needs and expectations between stakeholders, community members, and course requirements	*"It became very overwhelming at times to find myself immersed in [a] politically charged environment. What I took away from these stressful situations is how essential flexibility is moving forward in any collaborative public health project and how complex/delicate power can be. I learned of how my words via email or over the phone can be mistaken with some Yale baggage and as a result I am more mindful of the ways in which I choose to convey my thoughts, feelings, and intentions." (Student 4, 2015 Cohort)*
**Skills Learned**<br>Flexibility<br>Applying lessons from previous courses<br>Building project management and problem solving skills<br>Gaining experience in academic public health<br>Strengthening skills in qualitative and quantitative data analysis<br>Learning from teammates	*"[The preceptors] openly shared that going through evaluations and "crunching numbers" was not a current huge priority because of their huge workload. With this in mind, we had to consider a sustainable system that would not generate mountains more of paperwork, lead to resentment on the staff members' part of being given another item on their to-do list, or alienate participants in [the program] by making them feel forced to fill out a lengthy personal survey. These considerations led us to cut out huge portions of our original evaluations, including weight and diabetes status, and inspired us to think of efficient ways to administer surveys, like through QR codes." (Student 3, 2016 Cohort)*
**Personal Reflection**<br>Thinking critically about the tools used in public health research and practice<br>Reflections on the role of research in mitigating public health issues<br>Innovating new approaches to solving public health issues<br>Thinking about the importance of building and maintaining trust in academic-community partnerships	*"I began to question the outdatedness of the tools and frameworks we use today. This course has made me evaluate and re-evaluate certain norms and standards of practice in public health practice through various readings and class discussions." (Student 1, 2008 Cohort)*<br><br>*"Public health is about people, and interacting closely with [community members], as well as dealing with the issues that come with homelessness, helped me understand that there are stories behind every homelessness journal article and every case [organization] has dealt with. I learned just how important sensitivity is to both working in solidarity with the community being served and to having a real impact, a working with rather than a working for." (Student 3, 2016 Cohort)*
**Challenges**<br>Preceptors sometimes lacked the infrastructure and resources to support a student project<br>Internal politics/competing interests from within the organization<br>Time and resource limitations	*"The most difficult part for me personally was the fact that I did not realize how fragmented and disorganized the [community health worker (CHW)] network was in [Connecticut]. I did not know what I was getting myself into in terms of a lack of available individuals in the state to speak with for interviews. This has been a very overwhelming project as I feel that this lack of knowledge about the current CHW system in [Connecticut] has caused my group much confusion, especially in terms of identifying reimbursement mechanisms for CHWs as part of our project, which is up in the air with healthcare reform. This coupled with the short amount of time to turn over a report for class has been extremely overwhelming, however I have simultaneously learned a lot about how projects work in the real world; nothing is ever black and white, nor simple." (Student 1, 2013 Cohort)*
**Important Course Elements**<br>Strong mentorship and support from the preceptors and teaching team<br>Communication and respect<br>Recognition of individual strengths	*"We would sometimes leave a meeting confused by the aims of our project, which would facilitate needing to follow up with our preceptor. This, in particular, also helped me develop communication skills as we often had to ensure we were responding in a prompt and courteous manner. It was important that we continued to respect our preceptor's larger goals as we shaped this project. Our strong relationship with our preceptor would not have continued if we did not have this openness and respect for her ideas." (Student 5, 2018 Cohort)*
