# Supplementary material for: Action learning and public health pedagogy: Student reflections from an experiential public health course
Source: Front Public Health. 2023 Mar 28;11:1128705. doi: 10.3389/fpubh.2023.1128705 (PMC10086179; doi:10.3389/fpubh.2023.1128705)
Supplement: Supplementary file 1 [file Data_Sheet_1.ZIP › Supplementary Material Presentation/Appendix B - Assignment Instructions 2.pdf]

YSPH Public Health Practice Requirement Guidelines (2009-2015)	
1	Practicum may occur in a wide variety of settings at the local, regional, national or international level but must be outwardly focused on a public health problem or issue. Acceptable venues would include governmental entities, nongovernmental, and private sector organizations with a public health component such as pharmaceutical companies, hospitals, managed care/health maintenance organizations, and consulting firms.
2	Practicum affords opportunity to participate in the full spectrum of defining, analyzing and addressing a "real life" public health problem or issue, either directly or through observation, consultation with others working on problem, participation in relevant meetings or activities, and pertinent reading.
3	Practicum entails one or more of the following roles:
	Assessment, monitoring, and/or surveillance of population health indicators, social determinants of health, inequities associated with race/ethnicity and socioeconomic status, environmental/occupational hazards and exposures, and other public health issues;
	Participating in the development and/or execution of applied public health research in the biological, environmental and social/behavioral realms, that has an immediate impact on public health, including translational, evaluation and epidemiological research efforts that contribute to the evidence-base and efficacy of public health practice;
	Planning, designing, implementing and evaluating public health interventions;
	Developing disease prevention and health promotion, media advocacy or risk communication materials;
	Developing, implementing and evaluating public health laws, regulations and policy;
	Participating in administrative/management activities of governmental and non-governmental public health agencies and/or health service delivery systems such as hospitals or community health centers. Activities could include organizational analysis and restructuring processes, strategic and business planning, organizational policy and protocol, financial management, budgeting and reimbursement processes, preparation of internal or external reports, human resources management, workforce development and credentialing, and addressing regulatory compliance issues such as audits and accreditation processes;
	Supporting the development and goals of public health coalitions through community organizing and advocacy efforts, needs assessments, strategic and participatory community planning, leadership development, and assisting with the development and implementation of community health improvement plans that respond to local needs and priorities.
4	Practicum integrates public health theory, knowledge, and skills, and applies and reinforces the learning objectives in MPH course work.
5	Practicum aligns with the student's area of specialization.
6	The practicum project and student role are appropriate for the MPH level.
7	The practicum agency and preceptor have requisite population health orientation, public health expertise, and infrastructure to support MPH level student learning experience.
8	The practicum has deliverables of tangible value to the mission of the placement agency /site.

YSPH Core Public Health Competencies (2016-2017)	
1	Demonstrate a knowledge base in the disciplines of biostatistics, chronic and infectious disease epidemiology, health systems, public policy, social and behavioral sciences, and environmental health.
2	Apply basic research skills to specific public health problems in both group and individual settings, including the ability to define problems; construct, articulate and test hypotheses; draw conclusions; and communicate findings to a variety of audiences.
3	Explain the interrelationships between a multitude of factors that can impact on a public health problem, including scientific, medical, environmental, cultural, social, behavioral, economic, political, and ethical factors.
4	Review, critique, and evaluate public health reports and research articles.
5	Apply public health concepts, principles, and methodologies obtained through formal course work to actual problems experienced in the community or work environment.
6	Critically evaluate programs, interventions, and outcomes that relate to public health practice.
7	Apply ethical standards and professional values as they relate to the practice of public health.
8	Demonstrate sensitivity to the social context within which public health professionals practice.

YSPH Cross-Cutting Competencies (2016-2017)	
Communication and Informatics	Collect, manage and organize data to produce information and meaning that is exchanged by use of signs and symbols; to gather, process, and present information to different audiences in-person, through information technologies, or through media channels; and to strategically design the information and knowledge exchange process to achieve specific objectives.
Diversity and Culture	Interact with both diverse individuals and communities to produce or impact an intended public health outcome.
Leadership	The ability to create and communicate a shared vision for a changing future; champion solutions to organizational and community challenges; and energize commitment to goals.
Professionalism	Demonstrate ethical choices, values and professional practices implicit in public health decisions; consider the effect of choices on community stewardship, equity, social justice and accountability; and to commit to personal and institutional development.
Program Planning	Plan for the design, development, implementation, and evaluation of strategies to improve individual and community health.
Systems Thinking	Recognize system level properties that result from dynamic interactions among human and social systems and how they affect the relationships among individuals, groups, organizations, communities, and environments.

YSPH Core Public Health Competencies (2018)	
1	Demonstrate a knowledge base in the disciplines of biostatistics, chronic and infectious disease epidemiology, health systems, public policy, social and behavioral sciences, and environmental health.
2	Apply basic research skills to specific public health problems in both group and individual settings, including the ability to define problems; construct, articulate and test hypotheses; draw conclusions; and communicate findings to a variety of audiences.
3	Explain the interrelationships between a multitude of factors that can impact on a public health problem, including scientific, medical, environmental, cultural, social, behavioral, economic, political, and ethical factors.
4	Review, critique, and evaluate public health reports and research articles.
5	Apply public health concepts, principles, and methodologies obtained through formal course work to actual problems experienced in the community or work environment.
6	Critically evaluate programs, interventions, and outcomes that relate to public health practice.
7	Apply ethical standards and professional values as they relate to the practice of public health.
8	Demonstrate sensitivity to the social context within which public health professionals practice.
