# Supplementary material for: Action learning and public health pedagogy: Student reflections from an experiential public health course
Source: Front Public Health. 2023 Mar 28;11:1128705. doi: 10.3389/fpubh.2023.1128705 (PMC10086179; doi:10.3389/fpubh.2023.1128705)
Supplement: Supplementary file 1 [file Data_Sheet_1.ZIP › Supplementary Material Presentation/Revised Manuscript - 2.24.2022-tracked.docx]

**Action Learning and Public Health Pedagogy: Student Reflections from an Experiential Public Health Course**

**Christine N. Pham^1^, Shayna D. Cunningham^1,2^, Debbie L. Humphries^3*^**

^1^Department of Social & Behavioral Sciences, Yale School of Public Health, New Haven, CT

^2^Department of Public Health Sciences, UConn Health, Farmington, CT

^3^Department of Chronic Disease Epidemiology, Yale School of Public Health, New Haven, CT

*** Correspondence:**Debbie L. Humphries

Debbie.humphries@yale.edu

**Keywords (5-8):** community-based experiential learning, action learning, rapid qualitative analysis, rapid research, interprofessional education

**Abstract**

Introduction: Applied practice experiences are essential components of the Masters of Public Health (MPH) curriculum. The objective of this study was to examine students’ perspectives on the skills and expertise they developed in an MPH course offering applied practice opportunities.

Methods: Of 236 students who took the course from 2008-2018, email addresses were obtained for 212 and 104 completed the consent form. Following consent, reflection essays were de-identified and analyzed using a rapid qualitative analysis approach. The essays addressed students' learning experiences and application of the competencies for MPH programs set by the Council for Education in Public Health (CEPH). Deductive and inductive analytical lenses were used to identify the key lessons learned by each cohort of students. Semi-structured guides and matrixes for essay analysis were created using assignment instructions and CEPH competencies.

Results: Although the reflection paper assignment varied across the years, commonalities were observed in the student reflections. Key themes included turning theory into practice, navigating the complex environment of public health practice, skill building, critical self-reflection, challenges encountered, and elements that facilitated project success. Students reported developing practical skills, such as planning for independent research (eg., preparing for institutional review board (IRB) submission, consulting with faculty and other experts), identifying realistic approaches for data extraction during chart reviews and analyses of electronic medical records, and disseminating findings for diverse stakeholders and audiences. Students also reported strengthening cross-cutting skills such as communication, teamwork, and problem-solving that were useful for navigating power dynamics and balancing competing interests and expectations. Students explored their identity as public health professionals as they navigated the dynamics of public health practice.

Conclusion: The applied practice experience served as a valuable tool for knowledge and skills acquisition. Moreover, it served as an opportunity for students to engage with the unique organizational cultures of their respective community partners and to deepen their understanding the complexities of conducting meaningful community-engaged research.

Implications: This study demonstrates the utility of analyzing students’ critical self-reflections as a tool for exploring learning experiences when training future public health professionals. The findings can help educators design future applied practice experiences.

**1. Introduction**

Bridging the gap between research and practice is essential to addressing real-world public health issues. Experiential learning in conjunction with rigorous coursework has been shown to be a promising strategy for training students to translate academic research into praxis that will mitigate disparities and improve the health of diverse communities.(1,2) The Council for Education in Public Health (CEPH) is the accrediting body for public health training programs and seeks to provide a framework for public health education that is responsive to the needs of diverse communities.(3) In 2014, CEPH revised its accreditation requirements to emphasize a set of competencies that highlight the science of public health as well as the skills needed to practice public health.(4) Applied practice experiences allow students to immerse themselves in the practice of public health in a mentored environment.(5) Practice-based Community Health Research (PBCHR) is a practicum offered by the Yale School of Public Health (YSPH) and satisfies the requirements of the MPH Applied Practice Experience as defined by CEPH.(4) This is an elective course that provides a unique opportunity for students to strengthen their research skills while learning to work within the complex dynamics of public health practice. MPH students take PBCHR in the spring semester of their first or second year. There are no prerequisites other than being an MPH student or the permission of the instructor. This course has been described previously, and is centered on student teams working collaboratively over the spring semester with preceptors from host community organizations to complete projects proposed by the host organizations.(5)

Since 2008, the current course instructor has oriented the course to center the importance of community partnerships for student and community capacity building, emphasizing the role of individuals in the relevant communities as experts on their own lived experiences.(6) Engaging community agencies as active and equal partners creates space for the elevation of knowledge and ideas not always represented in health literature.(6–8) Previous research has often focused on capacity building as community empowerment and acquisition of new skills; however, increasing capacity of academic partners and MPH professionals to pursue authentic research partnerships is equally important.(9,10) Several pedagogical strategies exist for teaching this strengths-based approach to community health research; however, there is a gap in the literature on the impact of such teaching methods on public health students.(10–12)

To assess the impact of applied practice experiences in public health training, we wanted to understand how students perceived their experience working in a local, community-centered context. Previous studies have identified the potential for analysis of reflective essays to illuminate students’ meta-cognitive processes in experiential learning.(13,14) The combination of applied learning and critical reflection is essential to a continual process of thinking and doing.(13) Similarly, Ball et al’s framework for training educators to advance equity suggests that epistemological change is generated when learners are simultaneously 1) exposed to theoretical frameworks, 2) challenged with real-world problem solving, and 3) engaged in critical self-reflection.(15)

Thematic elements gleaned from these studies can help educators better understand how students construct meaning from their practicum and classroom experiences. This project involved qualitative analysis of reflective essays from an experiential public health course to identify key elements of the student learning experiences. In analyzing reflection papers submitted two-thirds of the way through the semester, we identified commonalities in learning experiences and course elements that students experienced as most impactful.

**2. Methods**

*2.1 Participants*

All students in the Practice Based Community Health Research (PBCHR) course were graduate students at the Yale School of Public Health (YSPH) or other Yale-affiliated school. Table 1 shows the YSPH departments represented in the study sample. Table 2 organizes the number of agencies and students represented in the sample by year. Records held by the Yale School of Public Health alumni office and online searches were used to obtain email addresses of PBCHR students between 2008-2018. 24 out of 236 former students did not have recorded email addresses and thus, were not contacted for participation. All former students for whom email addresses were obtained were contacted by the course’s instructor. The emailed invitation to participate included an overview of the proposed project as well as an information sheet about the study and a link to a Qualtrics survey where students indicated their consent to participate. Out of 212 emails that did not lead to a bounce-back error, 104 consents were obtained after two rounds of outreach.

**Table 1:** Participant characteristics by department affiliation

**Prior to 2017, SBS was a division within the CDE department*

**

**Table 2:** Participant characteristics by year

*2.2 Data Characteristics*

Reflection papers were written by students about two thirds through the semester. Although assignment details changed throughout the years, all students were asked to reflect on their learning experiences in the course. We noticed trends in students essays throughout the decade; however, the underlying drivers of these trends are uncertain due to the diversity of students who have taken the course in the past decade. For example, in 2008, the assignment instructions simply asked students to reflect on ideas raised in course readings and apply these themes to their own current community project or past work. Students were also asked to include reflections on one of the public health code of ethics guidelines. Beginning in 2009, these reflective essays shifted away from a more narrative style when students were asked to link their learning experiences in the course to guidelines dictated by the YSPH Public Health Practice Requirements.

*2.3 Data Analysis*

Rapid qualitative analysis was used to identify themes underlying student experiences. Rapid analysis methods have been extensively refined and adapted by researchers needing to analyze qualitative data rapidly without sacrificing richness in findings (16–19). Matrixes have been used to streamline the process of identifying themes, similarities, and differences between responses in qualitative research (20,21). The current study utilized templates and a matrix based on the YSPH Public Health Practice Requirement Guidelines (2008-2015) and the YSPH Core Public Health & Cross-Cutting Competencies (2016-2018).

Essays from consenting alumni were anonymized by the course instructor prior to analysis. First, essays were organized by cohort as the reflection paper assignment varied slightly across the years. See Appendix A and B for assignment instructions. Summary templates in Microsoft Word were created using deductively generated codes based on assignment objectives for each cohort. These templates standardized the summarizing process while allowing flexibility for differences in assignment instructions by year. Additional codes were generated as needed. Materials from each summary were then reviewed and transferred into a matrix in Microsoft Excel to organize quotes by cohort. Quotes of interest were transferred to a second Excel workbook to systematically analyze all data under umbrella themes. All essays were analyzed by CNP and DLH to identify a priori and emergent themes. CNP completed the course in 2021, and DLH is the course instructor. Weekly meetings were used to address questions and discuss the themes that were identified.

*2.4 Ethics Approval Statement*

This study was determined to be exempt from review by the Yale University Social Science, Behavioral, and Educational Research Institutional Review Board.

**3. Results**

Overall, differences in department representation between the group of students who consented to essay analysis and the overall sample were minimal (data not shown). Six major themes emerged upon analysis of student reflection papers: turning theory into practice, navigating the complex environment of public health practice, skills learned, personal reflections, challenges, and strengths. See Appendix C for additional quotes.

**3.1. Theme 1: Turning Theory into Practice**

Students were able to apply the theories and frameworks they had learned in the classroom to real world problems and strengthen skills relevant to their future careers. In addition, students found the experience of working on real projects that were timely and relevant to neighboring communities to be fulfilling. Many wrote about feeling motivated by the knowledge that their work could directly benefit the community members served by their preceptor organizations.

*“When we began this project I had already enrolled in many biostatistics courses for my MPH concentration. However, biostatistics courses tend to emphasize theory over practice, leaving me relatively skilled at reciting the calculus behind the relationships between survivor functions but not so clear on how to wrestle EMR data into a format to which I can apply those statistical tools.” (Student 2, 2016 Cohort)*

*“We felt like our project was worth something. We were reminded time and time again throughout our project, especially during difficult times when it seemed we would have no interviewees, that our project was important and that it was serving to “plant the seed” of a necessary endeavor that [preceptor] had been wanting to do on their own for a long time but they needed someone to collect the necessary information first.” (Student 1, 2014 Cohort)*

**3.2. Theme 2: Navigating the complex environment of public health practice**

Hands-on experiences exposed students to the dynamics of public health practice and allowed students to immerse themselves in the complex process of developing solutions to immediate issues. Several students reported feeling overwhelmed by the realization of how politics and social dynamics can impact the direction of their project.

*“I learned of how politics heavily surrounded our project in [community], and it became very overwhelming at times to find myself immersed in politically charged environment. What I took away from these stressful situations is how essential flexibility is moving forward in any collaborative public health project and how complex/delicate power can be. I learned of how my words via email or over the phone can be mistaken with some [institution] baggage and as a result I am more mindful of the ways in which I choose to convey my thoughts, feelings, and intentions.” (Student 1, 2015 Cohort)*

This complexity led some students to highlight the importance of interprofessional collaboration and communication. Students also learned to balance the different needs and expectations that may arise from within a single preceptor organization.

Power was a running theme throughout many of the essays. Students reflected on their position as outside researchers and specifically, as graduate students from a well-known educational institution. One student noted how their partnership with the community agency forced them to critically reflect on the relationships between their preceptor and other institutions.

*“Working with [agency] required an understanding of systems and the way that constructs of power inform the way community actors and organizations function at different levels in ways that both hinder and strive to promote equity.” (Student 1, 2018 Cohort)*

While unequal power dynamics were often cited as challenges, some students also recognized this as a sign of a deeper issue of mistrust between community members and academic research institutions.

*"The [student organizing the interviews] was also the holder of the keys, and because of this, I was seen as having power by the inmates...However, building trust and rapport with the inmates was crucial for successful execution of the study and I often felt I held a level of power that I didn’t deserve.” (Student 1, 2008 Cohort)*

These power dynamics were also evident in the community agencies’ hesitancy to criticize institutions, especially when the organizations are dependent on outside stakeholders for donations and financial support.

*“Cultural and political factors play into the donations [agency] receives from [institution]. [institution] donations are difficult to incorporate into [agency] meals, and often do not meet sanitation guidelines and must be discarded. [agency] has not addressed these issues with [institution] because their relationship is skewed, with [institution] having much more social and political power. Similarly, [agency] cannot do much to change the donations they receive from other organizations.” (Student 2, 2018 Cohort)*

Aside from differences in power, students also identified differences in value systems. The reflexivity demonstrated in these essays revealed ways in which the students realized that their perceptions of health may not always align with the values that communities deem important. In addition to becoming more sensitive to the social context of their project, students learned to pivot quickly to ensure that their project met their preceptors’ needs.

*“I’ll admit when I first started on this project I was concerned the intervention might be damaging to the kids’ self-esteem: who wants to discuss their weight in a group with a bunch of strangers? Immediately, however, I saw this was not the case. Our culture’s obsession with privacy makes group health care relatively unusual, yet in communities like [community], it is a reasonable and perhaps desirable option. It allows providers to reach more patients, it is cost effective for clinics, and it has the important potential to foster a sense of social support among individuals with similar health issues.” (Student 2, 2008 Cohort)*

**3.3. Theme 3: Skills Learned**

Students highlighted a myriad of research and project management skills including data analysis, problem solving, interviewing, conducting focus groups, communication, and teamwork. Skills relevant to academic public health were also noted as valuable lessons from the course. Students felt that the practicum-oriented curriculum allowed them to not only strengthen their skills in the field, but also learn how to disseminate their findings. Students who had not participated in formal public health research before especially appreciated guidance on the IRB process and poster preparation. Students also gained experience in developing program planning tools such as logic models, program theories, and budgets. Outside of strengthening existing skills, true collaboration with community partners allowed students to learn the real-life application of skills learned in class and thus bridge the gap between research and practice.

*“My group has worked to [develop] a program theory and logic model, which has allowed us to us to assess our short- and long-term project goals as well as the theoretical basis for our project to impact positive change within and beyond [community]. We have also enhanced several skills necessary for community health practicum work, including developing a project budget and completing an IRB application for the project. Additionally, by having guest speakers who are conducting community-based participatory research at Yale and within the New Haven community, this practicum has improved our knowledge of similar work within the field*.” *(Student 1, 2013 Cohort)*

Bridging this gap between research and practice came with some growing pains. As the students worked on their project, they realized that standard research practices may not be applicable to every public health program. One student described how her team had to adapt their initial recommendations to avoid overburdening the preceptor agency:

*“We had to consider a sustainable system that would not generate mountains more of paperwork, lead to resentment on the staff members’ part of being given another item on their to-do list, or alienate participants in [the program] by making them feel forced to fill out a lengthy personal survey. These considerations led us to cut out huge portions of our original evaluations, including weight and diabetes status, and inspired us to think of efficient ways to administer surveys, like through QR codes.” (Student 1, 2016 Cohort)*

Communication, leadership, and teamwork skills were also frequently discussed throughout student essays. Although there were clashes in different working and learning styles at times, students described how they learned to quickly resolve conflicts and avoid miscommunication.

*“I feel as if I’ve strengthened my group communication skills… I believe that the coordination that we’ve had to do as a group has been truly impressive, and I’m proud of us for being to find time to travel to [community] for meetings and interviews. It takes a surprising amount of effort to coordinate the schedules of five MPH students, but we were able to manage it. I’m more understanding of other people’s schedules and conflicts, and I feel as if I’m become a much more flexible person.” (Student 3, 2018 Cohort)*

**3.4. Theme 4: Personal Reflections**

In addition to reflecting on the impact of their project, students also acknowledged the ways in which they had benefited from existing relationships between course instructors and community agencies. The course’s experiential model allowed students to catch a glimpse of the reality of public health practice. Some admitted that it was only after intimately working with community organizations that they learned to appreciate the unique strengths of these agencies - traits that may have been overlooked in the literature.

*“[agency] epitomizes a complex adaptive system, with changing external factors in the form of donation sources from foodbanks or institutions. Prior to this class I would have deemed [agency] as chaotic, an unproductive surface-level conclusion that connotes a certain inability for change. Now I would characterize [agency] as dynamical and uncertain, which indicates a different set of expectations and approaches.” (Student 1, 2018 Cohort)*

After working so closely with a community partner over the span of 4-5 months, many used this opportunity to reflect on their roles as researchers and think critically about the tools and metrics they had been taught to use. Some questioned the impact of their work as researchers and future public health professionals - there was a question of *how* they should conduct research instead of simply *if* they should research.

*“As a researcher I suppose the question to grapple with is whether you should do research to collect descriptive statistics alone, or if you should go one step further to help solve the problem; I think that the latter is the only way to make an impact.” (Student 3, 2008 Cohort)*

Time and resource constraints forced students to be creative and utilize novel approaches to solving public health issues. Upon reflecting on their experience working within these bounds, one student described changes in the way that they conceptualized problems and analyzed data.

*“I feel that this practicum experience has also introduced me to new methods and theories of ways to analyze data, and even consider what “data” is and how research questions can be derived from within the community for tangible and practical needs.” (Student 2, 2015 Cohort)*

**3.5. Theme 5: Challenges**

Challenges were commonly associated with differing expectations, limited support from preceptors, unfamiliarity with research practices, and time and resource constraints. Differing expectations between teams and preceptor organizations led to confusion and delayed progress. Sometimes, the public health issues that preceptors wished to address were too large to accomplish in one semester. Honest conversations between preceptors and student teams made it possible to determine more specific and manageable goals. One student explained:

*“Through multiple preliminary conversations and meetings with stakeholders, we were able to determine which programs to focus on and which health outcomes [agency] as a whole was most interested in assessing. In that way, we “defined the issue” to analyze and address. I found this part of the process the most challenging, since each staff member expressed different priorities and even different target groups. It certainly provided practical experience in working and communicating with a diversity of stakeholders.” (Student 3, 2015 Cohort)*

Although preceptors and project teams were eager to collaborate, time and resource limitations proved to be another common challenge.

*“They were really hoping for our team to evaluate almost every aspect across the organization including the community programs, urban farm, and high school. We had to communicate to them that we did not have the time or capacity to do this, and that we could focus on a few priorities in order to do a thorough job.” (Student 4, 2015 Cohort)*

In some cases, preceptors did not have the infrastructure to support a research-based community health project. Students found it difficult to seek guidance when the community agency itself was facing management issues. Administrative delays and unfamiliarity with research practices could also hinder progress. Some expressed the need for preceptors to undergo basic research ethics training to avoid setbacks related to IRB non-compliance.

Some teams struggled to design their project in a way that best fit the community partner’s needs while balancing competing interests from within the preceptor organization. Internal politics were difficult to navigate but proved to be valuable learning experiences in inter-professional collaboration.

*“A lack of communication between [two preceptors] required us to negotiate the organizational politics and hierarchy. This is the type of work that I can read about all day long, yet is essentially trial by fire in terms of internalizing the concepts and testing the practice. Overall, I strengthened my knowledge and capacity of developmental evaluation principles and CBPR values through being a part of the [agency] team.”* (*Student 1, 2018 Cohort)*

**3.6. Theme 6: Important Course Elements**

Adequate *support* from both stakeholders and faculty members can help teams overcome communication challenges. While this student previously noted communication challenges between their team and the community partner, they were able to move forward with the help of their preceptor. Although this preceptor did not consider themselves a public health expert, the team found their insight and support extremely valuable:

*“To my knowledge, I do not think any of the staff at [agency] have Public Health degrees or if they would consider themselves to have public health expertise. However, [the] agency and [preceptor] provided more support than I originally expected. He was very thoughtful in providing a theory of change and a logic model to us at the beginning of the course and continued to have excellent input on the content of the survey and the focus group questions.” (Student 3, 2015 Cohort)*

Many teams also found it helpful to *consult with experts* such as faculty members and representatives from organizations outside of their partnership. Mutual *communication and respect* were key elements of a positive relationship between preceptors and student teams.

*Gratitude* also went a long way - students described how showing appreciation for one another and recognizing each team member’s unique strengths helped them form tighter bonds. Many students found it helpful to set up informal gatherings with their peers before the course began.

*“Before the fall semester ended, we met up to create guidelines and expectations for the project and communication. Being able to step and delegate tasks is also very important. Having these clear guidelines allowed for the project to run smoothly. In previous group projects, there was often miscommunication. Meeting regularly with the preceptors also allowed for us to have clear communication with the lead center. We also discussed our expectations and learned about our preceptor’s expectations.” (Student 4, 2018 Cohort)*

**3.7. Other Observations**

Following a change in the assignment instructions in 2009, essays began to resemble a list of competencies they had addressed through their project. While this yields valuable information about the concrete skills that students identify as important, it limits the educator’s view of the student’s processes of identity construction throughout the course. However, several of the essays from 2009 onwards did include discussions of the personal lessons that students had learned by building authentic relationships with their preceptors. Probing for more personal reflection may make visible the factors that shaped students’ beliefs and values as future public health professionals.

**4. Discussion**

Innovations in public health pedagogy have highlighted the value of learning experiences where students are challenged to apply their public health skills in less controlled and more ‘real world’ environments.(22) Applied practice experiences can allow students to master concrete research skills that are crucial in developing a well trained public health workforce.(23,24) In 2011-2012, a survey launched by the New York City Health Department examined the most common skill weaknesses among MPH graduates working in local health departments. These included quantitative data analysis, scientific writing, and project management.(25) Like other applied practice courses described in the literature, CBPHR gave students the opportunity to practice synthesizing and applying evidence-based knowledge to real world problems.(24,26–29) Many students reported honing skills that they had not previously developed such as submitting Institutional Review Board (IRB) applications, extracting and cleaning data from electronic medical records, and preparing research posters. Team-based learning during the semester project allowed students to collaborate with peers from different training backgrounds and gave them the opportunity to strengthen less developed research skills.(30)

Communication, leadership, research, and project management were reported to be some of the most valuable cross-cutting skills learned from the course. Despite taking rigorous research methods courses in the past, a number of students reflected on the difficulties they faced putting these skills into practice. Similar to students from other practice-based public health courses, students in this study noted challenges related to the length of the course.(12,31) A single semester left students with a relatively short time frame to adapt and pivot as needed when they were inevitably faced with unexpected challenges and roadblocks. Despite these challenges, many students wrote about their personal growth through the semester. While many students described themselves as “type-A” individuals who have a natural tendency to take on leadership roles in the past, the collaborative nature of the course forced them to quickly adapt to each other’s working styles. Building close relationships with their preceptors allowed for students to become embedded in the culture of their preceptor organization - giving them another perspective of the reality of public health practice. Working with community partners challenged students to apply their research skills in a way that was sensitive to the political and economic context of the community. For many, problem solving involved more than just building a good team dynamic. It also required students become comfortable working in ambiguity and to be diligent in making sure that their project met the interests and needs of their preceptors. These findings are consistent with previous research on experiential course design for clinical students.(14,32)

In this course, learning occurred through meaningful collaboration between students and their preceptors. Although some students entered the course with background knowledge of community health, many wrote about how their project experiences allowed them to gain a deeper understanding of public health practice. Several students grappled with the power imbalance that is inherent in many community research partnerships. Additionally, some students addressed their positionality as students from a prestigious institution with a deep and complex history with the surrounding community.(33) While this study did not explicitly investigate how students perceived their roles as “outsiders” in their research project, reflections on power imbalances came up in multiple student essays. This is another gap in the research on student experiences in public health pedagogy. Previous studies have utilized qualitative methods to analyze perceptions of power in community-academic research partnerships but these are limited in number and often do not focus on the perspective of students or trainees.(34–36) Several students in this study reflected on how their experiences within the course exposed them to the realities of practicing public health at a community level. Previous studies have suggested that active learning via community partnerships can encourage students to foster a sense of civic responsibility.(24) Many students who did not plan on pursuing careers in community health reported feeling inspired to incorporate principles for community-engaged research into their future work.(37) While a curriculum focused on career readiness and skill mastery is important for upholding practice standards and promoting innovation in research, there is a continued need for innovation in public health pedagogy to drive social change and address health inequities.(38,39) Thus, the inclusion of required readings and lectures on theoretical frameworks in public health practice could have helped prepare students for their participation in community health research while encouraging them to develop their worldview.(40)

Although these field settings are essential for preparing students for their future careers as public health professionals, applied practice experiences such as this course require a heavy amount of preparatory work. While there was great enthusiasm from students to be in partnership with community organizations, any university-community dyad demands a great deal of conscientiousness and diligence from the teaching team to ensure that preceptor organizations benefit from their involvement in students’ education.(41) The instructor(s) and teaching assistants needed to make sure that the scale and scope of each project matched the skillsets, availabilities, and interests of students.(31) Key elements for success included: support from preceptors and course instructors, clear communication between team members and community partners, coursework about topics relevant community health practice, and guidance on program planning tools.(42) Students commonly reported the importance of having strong support and guidance from preceptors and course instructors. These findings were consistent with lessons described by instructors and students of applied practice courses at different public health programs.(28,31) An article evaluating an experiential learning program at the University of Iowa College of Public Health noted the importance of including a preparatory classroom component to the applied practice experience.(28) This recommendation was echoed by several CBPHR students who reported feeling more confident in their research and project management skills because of the instruction they had received in weekly lectures and reading assignments. Challenges associated with conflicting expectations between stakeholders suggest a need for processes to determine preceptor and student readiness before collaboration can begin.

For many, this “hands on'' experience allowed them to see the connection more clearly between big data and lived experiences.(43,44) Reflections from student essays demonstrated the value of experiential learning in exposing students to the different ways in which they can apply their existing skills to problems addressed by local service organizations. Especially for students who did not plan on staying in the realm of community health research or practice, experiential learning in this context was a rare opportunity to directly engage with the people affected by public health issues that they may address later in their careers.

**4.1. Implications**

Results from this study can help educators better understand students’ perspectives on experiential learning and inform the development and evaluation of other applied practice courses in public health education. In addition to revealing key elements of the course that students found important, rapid analysis of student reflection essays demonstrated the value of essay analysis as a tool for exploring students’ learning experiences. Leveraging reflective practices is crucial for examining how experiential learning can play a role in developing students’ professional identity as well as understanding and passion for public health.(45,46) Analyzing student reflections from applied practice courses through a social justice lens may reveal the thought processes that occur as students observe how health inequities manifest in the specific context of their project. Encouraging reflection throughout the course may not only provide narratives for future analyses but also encourage students to critically examine their role as future public health practitioners.(12,31)

**4.2. Limitations**

While reflection papers were always due 2-3 weeks after spring break, not all teams were on the same stages of their project at this point in the semester. This discrepancy may explain slight variances in student reflections because not all students had finished their project at the time their essays were written. Class sizes varied, and more recent student cohorts were larger. Students from the 2015 through 2018 classes contributed 50% of the essays analyzed; thus, results may not be representative of student experiences from earlier years. Inability to contact students from earlier years may explain differences in response rates. Furthermore, variances in response rates among cohorts may obscure themes that are unique to certain groups of students. Because this study includes the analysis of essays written over the span of 11 years, student experiences may differ due to changing social contexts and emerging theories in public health research.

Although this study was able to capture immediate reflections, its retrospective design limits our understanding of the processes by which students construct meaning from the relationships they built with their peers and community partners. Beliefs and attitudes formed during the years after the course were not included in this study. Longitudinal investigation of alumni experiences would be needed to understand the longer-term impacts of the course. Essays included in this study predate the COVID-19 pandemic, so some findings may not be generalizable for experiential public health courses after March 2020. Adapting to a world where COVID-19 remains a threat would require flexibility to accommodate for students’ needs while being mindful of budget and resource constraints on community organizations.(11)

**5. Conclusion**

Experiential learning in public health provides students with a unique opportunity to collaborate with local community organizations and directly engage in public health practice. The themes identified in this study reveal lessons learned and challenges faced by students in the Practice-based Community Health Research course. Student reflections also revealed key elements of the course that facilitated success in their projects. Observations made throughout the course helped shape students’ beliefs and values about public health, which in turn, will likely influence the approaches that they take in their future work. The methods used demonstrate potential for harnessing reflective practices in teaching and understanding students’ learning experiences.

**6. Conflict of Interest**

The authors declare that the research was conducted in the absence of any commercial or financial relationships that could be construed as a potential conflict of interest.

**7. Acknowledgements**

The authors would like to thank the former PBCHR students for participating in this study and sharing their narratives. The content of this manuscript has been presented in part online within the first author's Master’s thesis.(47)

**8. References**

1. Marriott LK, Lipus AC, Choate L, Smith J, Coppola L, Cameron WE, et al. Experiential Learning Through Participatory Action Research in Public Health Supports Community-Based Training of Future Health Professionals. Pedagogy Health Promot. 2015 Dec 1;1(4):220–32.

2. Woods-Jaeger B, Daniel-Ulloa J, Kleven L, Bucklin R, Maldonado A, Gilbert PA, et al. Building Leadership, Capacity, and Power to Advance Health Equity and Justice through Community-Engaged Research in the Midwest. Am J Community Psychol. 2021 Mar;67(1–2):195–204.

3. Council on Education for Public Health Accreditation Criteria [Internet]. [cited 2021 Nov 2]. Available from: https://media.ceph.org/wp_assets/2016.Criteria.pdf

4. Foster A, King LR, Bender K. Are Public Health Academia, Professional Certification, and Public Health Practice on the Same Page? J Public Health Manag Pract. 2018 May;24(3):S47–50.

5. Humphries DL, Bastida C, Walker MC. Lessons From a Community Driven Experiential Public Health Course. Pedagogy Health Promot. 2021 Nov 11;23733799211056310.

6. Filler T, Benipal PK, Torabi N, Minhas RS. A chair at the table: a scoping review of the participation of refugees in community-based participatory research in healthcare. Glob Health. 2021 Dec;17(1):103.

7. Culhane-Pera KA, Pergament SL, Kasouaher MY, Pattock AM, Dhore N, Kaigama CN, et al. Diverse community leaders’ perspectives about quality primary healthcare and healthcare measurement: Qualitative community-based participatory research. Int J Equity Health. 2021 Dec;20(1):226.

8. Han HR, Link to external site this link will open in a new window, Xu A, Mendez KJW, Okoye S, Cudjoe J, et al. Exploring community engaged research experiences and preferences: a multi-level qualitative investigation. Res Involv Engagem. 2021;7:1–9.

9. Hicks S, Duran B, Wallerstein N, Avila M, Belone L, Lucero J, et al. Evaluating community-based participatory research to improve community-partnered science and community health. Prog Community Health Partnersh Res Educ Action. 2012;6(3):289–99.

10. Rubin CL, Martinez LS, Chu J, Hacker K, Brugge D, Pirie A, et al. Community-Engaged Pedagogy: A Strengths-Based Approach to Involving Diverse Stakeholders in Research Partnerships. Prog Community Health Partnersh Res Educ Action. 2012;6(4):481–90.

11. Mejia AP. “Plan for the Worst, Hope for the Best, but Realistically, Expect a Combination of Both”: Lessons and Best Practices Emerging from Community-Engaged Teaching During a Health Crisis. J High Educ Outreach Engagem [Internet]. 2021 Sep 22 [cited 2021 Nov 3];25(3). Available from: https://openjournals.libs.uga.edu/jheoe/article/view/2556

12. Kelley A. Critical reflections from a community-based participatory research course. Educ Health Abingdon Engl. 2013;26(3):178–82.

13. Ash SL, Clayton PH. Generating, Deepening, and Documenting Learning: The Power of Critical Reflection in Applied Learning. 2009 [cited 2022 Apr 27]; Available from: https://scholarworks.iupui.edu/handle/1805/4579

14. Kelly L, Walters L, Rosenthal D. Community-based medical education: is success a result of meaningful personal learning experiences? Educ Health Abingdon Engl. 2014 Apr;27(1):47–50.

15. Ball AF, Greene DM, Friedman JSL, Dray BJ. The Trifecta Framework: Preparing Agents of Change in Urban Education. Urban Educ. 2021 Jun 17;004208592110231.

16. Beebe J. Rapid Qualitative Inquiry: A Field Guide to Team-Based Assessment. Rowman & Littlefield; 2014. 283 p.

17. Sangaramoorthy T, Kroeger KA. Rapid Ethnographic Assessments: A Practical Approach and Toolkit For Collaborative Community Research. London: Routledge; 2020. 198 p.

18. Vindrola-Padros C, Johnson GA. Rapid Techniques in Qualitative Research: A Critical Review of the Literature. Qual Health Res. 2020 Aug 1;30(10):1596–604.

19. Watkins DC. Rapid and Rigorous Qualitative Data Analysis: The “RADaR” Technique for Applied Research. Int J Qual Methods. 2017 Dec 1;16(1):1609406917712131.

20. Averill JB. Matrix analysis as a complementary analytic strategy in qualitative inquiry. Qual Health Res. 2002 Jul;12(6):855–66.

21. Kara A, Wright C, Funches L, Williamson F, Hicks RA, Sutton TA, et al. Serving on a Graduate Medical Education Diversity, Equity, Inclusion, and Justice Committee: Lessons Learned From a Journey of Growth and Healing. Front Public Health. 2022 Apr 27;10:867035.

22. Meredith GR, Welter CR, Risley K, Seweryn SM, Altfeld S, Jarpe-Ratner EA. A New Baseline: Master of Public Health Education Shifting to Meet Public Health Needs. J Public Health Manag Pract JPHMP. 2022 Oct 1;28(5):513–24.

23. Caron RM, Hiller MD, Wyman WJ. Public health system partnerships: role for local boards of health in preparing the future public health workforce. J Community Health. 2014 Feb;39(1):29–34.

24. Yigletu S, Kosinski KC, Kuah A, Alfaro K, Holmes AC, Tendulkar SA. “Collaboration Toward One Collective Goal”: A Mixed-Methods Study of Short-Term Learning Outcomes and Long-Term Impacts Among Students Participating in an Undergraduate Community-Based Participatory Research (CBPR) Course. Front Public Health. 2021 Jun 21;9:694840.

25. Hemans-Henry C, Blake J, Parton H, Koppaka R, Greene CM. Preparing Master of Public Health Graduates to Work in Local Health Departments. J Public Health Manag Pract JPHMP. 2016;22(2):194–9.

26. Minlikeeva AN, Amato KA, Przybyla SM. Modern public pealth problems and solutions: An undergraduate capstone course to prepare the next generation of public health practitioners to enhance health equity. Front Public Health [Internet]. 2022 [cited 2022 Nov 28];10. Available from: https://www.frontiersin.org/articles/10.3389/fpubh.2022.992835

27. Martinez LS, Perea FC, Ursillo A, Pirie A, Ndulue UJ, Oliveira C, et al. Research as Curriculum: Engaging Undergraduates and Community Residents in Immigrant Health Research Partnerships. Prog Community Health Partnersh. 2012 Winter;6(4):491–8.

28. Chorazy ML, Klinedinst KS. Learn by Doing: A Model for Incorporating High-Impact Experiential Learning Into an Undergraduate Public Health Curriculum. Front Public Health. 2019 Feb 26;7:31.

29. Villanueva AM, Hovinga ME, Cass JL. Master of Public Health Community-Based Practicum: Students’ and Preceptors’ Experiences. J Public Health Manag Pract. 2011 Aug;17(4):337–43.

30. Lang DL, Walker ER, Steiner RJ, Woodruff RC. Implementation and Mixed-Methods Evaluation of Team-Based Learning in a Graduate Public Health Research Methods Course. Pedagogy Health Promot. 2018 Jun 1;4(2):140–50.

31. Flicker S, Mawani FN, Dellavilla M. Reflections on Teaching, Learning and Doing Participatory Research in a Graduate Seminar. Prog Community Health Partnersh. 2019 Fall;13(3):293–302.

32. Takamura A, Misaki H, Takemura Y. Community and interns’ perspectives on community-participatory medical education: From passive to active participation. Fam Med. 2017;49(7):507–13.

33. Hogan A, Panos J. Yale University and the New Haven Community [Internet]. ArcGIS StoryMaps. 2021 [cited 2022 Dec 13]. Available from: https://storymaps.arcgis.com/stories/615c749ffb1c4b8a823648bb46bc37aa

34. Caldwell WB, Reyes AG, Rowe Z, Weinert J, Israel BA. Community Partner Perspectives on Benefits, Challenges, Facilitating Factors, and Lessons Learned from Community-Based Participatory Research Partnerships in Detroit. Prog Community Health Partnersh Res Educ Action. 2015;9(2):299–311.

35. Jagosh J, Bush PL, Salsberg J, Macaulay AC, Greenhalgh T, Wong G, et al. A realist evaluation of community-based participatory research: partnership synergy, trust building and related ripple effects. BMC Public Health. 2015 Jul 30;15:725.

36. Egid BR, Roura M, Aktar B, Amegee Quach J, Chumo I, Dias S, et al. ‘You want to deal with power while riding on power’: global perspectives on power in participatory health research and co-production approaches. BMJ Glob Health. 2021 Nov 11;6(11):e006978.

37. Kim MM, Cheney A, Black A, Thorpe RJ, Cene CW, Dave GJ, et al. Trust in Community-Engaged Research Partnerships: A Methodological Overview of Designing a Multisite Clinical and Translational Science Awards (CTSA) Initiative. Eval Health Prof. 2020 Sep;43(3):180–92.

38. DeSalvo KB, Wang YC, Harris A, Auerbach J, Koo D, O’Carroll P. Public Health 3.0: A Call to Action for Public Health to Meet the Challenges of the 21st Century. Prev Chronic Dis. 2017 Sep 7;14:E78.

39. Merzel CR. Pedagogy to Guide the Next 70 Years of SOPHE and 150 Years of American Public Health. Pedagogy Health Promot. 2022 Dec 1;8(4):243–5.

40. James TG, Sullivan MK, Henderson H, Varnes JR. Competency Focused Versus Philosophically Grounded Health Promotion Practice: Impacts on Innovation and Addressing Health Inequities. Pedagogy Health Promot. 2022 Dec 1;8(4):246–50.

41. Tryon EA. Learning and Teaching Community-Based Research: Linking Pedagogy to Practice. J High Educ Outreach Engagem. 2015 Jun 23;19(2):201–6.

42. DRAHOTA A, MEZA RD, BRIKHO B, NAAF M, ESTABILLO JA, GOMEZ ED, et al. Community‐Academic Partnerships: A Systematic Review of the State of the Literature and Recommendations for Future Research. Milbank Q. 2016 Mar;94(1):163–214.

43. Soliman AS, Stainton L, Chamberlain RM. Experiential Learning in Career Development. J Cancer Educ. 2021 Aug 1;36(4):874–9.

44. Ruckenstein M, Schüll ND. The Datafication of Health. Annu Rev Anthropol. 2017;46(1):261–78.

45. Blenner SR, Roth SE, Manukyan R, Escutia-Calderon Y, Chan-Golston AM, Owusu E, et al. Community Partnerships and Experiential Learning: Investing in the Next Generation of a Diverse, Qualified Public Health Workforce. Pedagogy Health Promot. 2021 Dec 1;7(1_suppl):51S-62S.

46. Kumagai AK, Naidu T. Reflection, Dialogue, and the Possibilities of Space: Acad Med. 2015 Mar;90(3):283–8.

47. Pham C. Exploring Learning Experiences Of Students In An Experiential Community Health Practice Course. Public Health Theses [Internet]. 2022 Jan 1; Available from: https://elischolar.library.yale.edu/ysphtdl/2189
